# The Influence of COVID-19 on Antimicrobial Resistance Trends at a Secondary Care Hospital in Slovenia: An Interrupted Time Series Analysis

**DOI:** 10.3390/antibiotics13111033

**Published:** 2024-11-01

**Authors:** Samo Jeverica, Darja Barlič Maganja, Jani Dernič, Peter Golob, Alenka Stepišnik, Bojan Novak, Lea Papst, Anamarija Juriševič Dodič, Mladen Gasparini

**Affiliations:** 1 Izola General Hospital, 6310 Izola, Slovenia; jani.dernic@sb-izola.si (J.D.); peter.golob@sb-izola.si (P.G.); alenka.stepisnik@sb-izola.si (A.S.); bojan.novak@sb-izola.si (B.N.); mladen.gasparini@sb-izola.si (M.G.); 2 Faculty of Health Sciences, University of Primorska, 6000 Koper, Slovenia; darja.maganja@fvz.upr.si; 3 Department of Infectious Diseases, University Medical Centre Ljubljana, 1000 Ljubljana, Slovenia; lea.papst@kclj.si; 4 Department of Medical Microbiology Koper, National Laboratory of Health, Environment and Food, 2000 Maribor, Slovenia; anamarija.jurisevic.dodic@nlzoh.si

**Keywords:** antimicrobial resistance, COVID-19, Slovenia, interrupted time series analysis

## Abstract

**Background/Objectives.** Our study aimed to determine the development of antibiotic resistance during the peri-pandemic period in a regional secondary care hospital using an interrupted time series analysis. **Methods**. We analyzed data from seven years, accounting for 441,149 patient days. The incidence density of multidrug-resistant bacteria (MDR) burden and infection was reported per 1000 patient days. **Results.** During the COVID-19 period, a significant increase in the mean incidence density of the total MDR burden from 4.93 to 5.81 per 1000 patient days was observed (*p* = 0.007). On the other hand, the mean incidence density of MDR infections decreased from 1.61 to 1.29 per 1000 patient days (*p* = 0.019). Using the interrupted time series analysis, the same trends were observed, namely the overall increasing trend in MDR burden and the overall decreasing trend in MDR infections. This divergent trend is mainly due to similar trends in several Gram-negative MDR, namely ESBL-EC, ESBL-KP and CRE. **Conclusions.** Due to the increasing burden of MDR, it is necessary to strengthen AMR surveillance. In addition, strict infection prevention and control measures, and antimicrobial stewardship programs continue to be important components in the fight against resistant bacteria.

## 1. Introduction

Antimicrobial resistance (AMR) is one of the greatest public health and economic challenges of our time [1,2]. It was recently estimated that in 2021, 4.71 million deaths were associated with bacterial AMR and 1.14 million died directly from AMR [3]. The problem is particularly serious in the elderly population, with AMR increasing by 80% in people over the age of 70. Worldwide, methicillin-resistant *Staphylococcus aureus* (MRSA) remains the deadliest resistant pathogen, while carbapenem resistance in Gram-negative bacteria has increased the most in the last 30 years [3]. AMR is a global problem that affects all geographic regions and all socio-economic groups [2,3]. The World Bank predicts that up to 3.8% of global GDP could be lost to AMR by 2050 [4]. As antibiotic resistance continues to increase, humanity is in danger of losing many of the benefits that antibiotics have brought to medicine, both in the treatment and prevention of infections.

The COVID-19 pandemic was one of the biggest global crises in recent history [5]. To date, the World Health Organization (WHO) reported over 7 million deaths from COVID-19 since the start of the pandemic in 2019. However, it is estimated that the excess mortality associated with the pandemic is up to 2.74 times higher than the reported cases [6]. In Slovenia, the first case of infection was detected on 4 March 2020 and the epidemic was declared on 12 March 2020. The first wave (spring 2020) was mild compared to other European countries and the number of daily positive PCR tests never exceeded 61 [7]. The second wave began in September 2020 and lasted largely until July 2021, when the introduction of the Omicron variant initiated the third wave, which lasted until 2022. By March 2023, 9201 people had died from COVID-19 in Slovenia [8].

During the pandemic, healthcare systems worldwide faced the challenges of a shortage of medical staff, COVID-19-specific regular and, in particular, intensive care beds, and personal protective equipment (PPE) as well as several knowledge gaps. The high use of antibiotics in patients with COVID-19 pneumonia combined with gaps in treatment measures threatened to exacerbate the AMR problem in some clinical settings [9,10].

The aim of our study was to determine the evolution of antibiotic resistance during the peri-pandemic period in a regional secondary care hospital and to compare two quasi-experimental statistical methods for the comparison of AMR between two time periods.

## 2. Results

**Figure 1:**
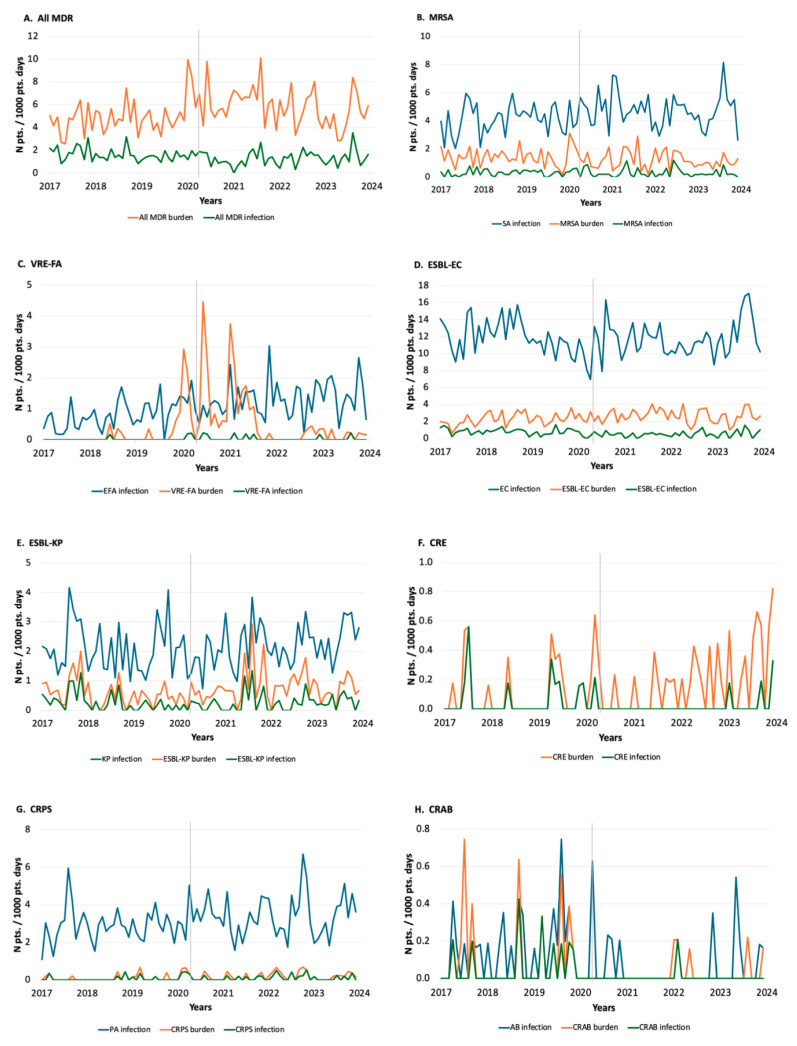
The incidence density of MDR burden, MDR infections and infections with specific organisms, where applicable, in the Izola General Hospital between 2017 and 2023. The vertical line delineates the period before COVID-19 and during COVID-19. (Abbreviations for multidrug-resistant bacteria are the same as in the text; pts. patients; burden refers to incident cases or organisms in clinical and surveillance cultures per patient; infections refer to incident cases of organisms in clinical cultures per patient).

We analyzed data from across seven years, accounting for 441,149 patient days (range: 55,986–67,527 per year). The incidence densities of multidrug-resistant bacteria (MDR) burden, MDR infections and infection with corresponding bacteria during the study period are shown in Figure 1. Two main patterns can be observed among the studied bacteria over time. The first group of bacteria, which includes vancomycin-resistant *Enterococcus faecium* (VRE-FA) (Figure 1C), extended-spectrum beta-lactamase-producing *Klebsiella pneumoniae* (ESBL-KP) (Figure 1E) and carbapenem-resistant *Acinetobacter baumannii* (CRAB) (Figure 1H), showed an inconsistent pattern over time. For VRE-FA, a general increase in *E. faecium* infections can be observed throughout the study period. At the same time, this was interrupted between 2019 and 2021 by a sharp rise in VRE-FA burden, which was not reflected in VRE-FA infections. Similarly, there were at least two periods where ESBL-KP burden and possibly ESBL-KP infections increased both before and during COVID-19. And finally, the incidence of *A. baumannii* infections and CRAB burden and infections all declined after 2020. The second group of bacteria, such as MRSA (Figure 1B), extended-spectrum beta-lactamase-producing *Escherichia coli* (ESBL-EC) (Figure 1D), carbapenem-resistant Enterobacteriaceae (CRE) (Figure 1F) and carbapenem-resistant *Pseudomonas aeruginosa* (CRPS) (Figure 1G), showed a more consistent pattern of incidence dynamics over time in both the increasing and decreasing directions. Most concerning is the steady increase in CRE burden over the years, with more frequent and higher peaks on the graph. Fortunately, this has not translated into more CRE infections over time. The steady decline in MRSA and ESBL-EC infections is also encouraging.

A comparison of incidence density of MDR burden and infections between the pre-COVID-19 and COVID-19 periods is shown in Table 1. In the COVID-19 period, a significant increase in the mean incidence density of the total MDR burden from 4.93 to 5.81 per 1000 patient days was observed (*p* = 0.007). A similar increase was observed for most of the specific MDR monitored, with the exception of MRSA and CRAB, where a decrease in mean incidence density was observed. On the other hand, the mean incidence density of MDR infections decreased from 1.61 to 1.29 per 1000 patient days (*p* = 0.019). This overall decrease was due to the significant reduction in ESBL-EC, CRE and CRAB infections.

**Figure 2:**
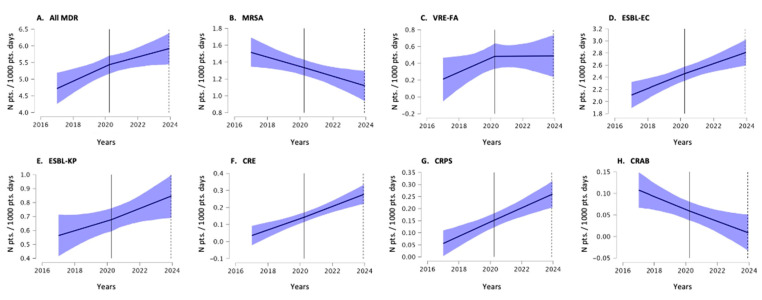
Trends in MDR burden in the Izola General Hospital between 2017 and 2023, determined using an interrupted time series analysis. The blue area shows the 95% confidence interval of the trend. The vertical line delineates the period before COVID-19 and during COVID-19. (Abbreviations for multidrug-resistant bacteria are the same as in the text; pts. patients).

**Figure 3:**
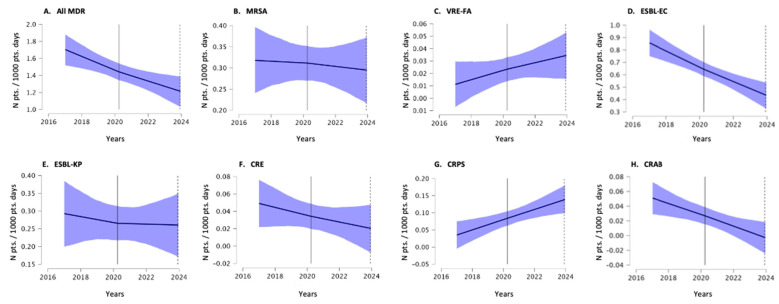
Trends in MDR infections at the Izola General Hospital between 2017 and 2023, determined using an interrupted time series analysis. The blue area shows the 95% confidence interval of the trend. The vertical line delineates the period before COVID-19 and during COVID-19. (Abbreviations for multidrug-resistant bacteria are the same as in the text; pts. patients).

The trends in the incidence density of the MDR burden and MDR infections, which were determined using the interrupted time series analysis, are shown in Figure 2 and Figure 3. In this more dynamic representation, the same trends were observed, namely the overall increasing trend in MDR burden and the overall decreasing trend in MDR infections. The corresponding offset, the growth coefficient in the pre-COVID-19 period and the change in growth during the COVID-19 period are shown in Table 1.

## 3. Discussion

We analyzed the dynamics of antimicrobial resistance among the most common MDR in the peri-COVID-19 period in a secondary care teaching hospital with a low burden of COVID-19-related hospitalizations. We found mixed patterns of evolution of MDR burden and MDR infections over time. Overall, MDR burden incidence density significantly increased (from 4.93 to 5.81 per 1000 patient days), while MDR infection incidence density significantly decreased (1.61 to 1.29 per 1000 patient days) over the study period. This discrepant trend was mostly due to similar trends in several Gram-negative MDR, namely ESBL-EC, ESBL-KP and CRE.

The data are reassuring on the one hand and may reflect our hospital’s restrictive policy on the use of antimicrobials both before and during COVID-19. Our multimodal antimicrobial stewardship program includes local guidelines for empirical antibiotic treatment, a restrictive list of antibiotics and a regular review of patients receiving antibiotics by both the infectious disease specialist on call and the clinical pharmacist. In addition, the hospital’s COVID-19 management team developed a restrictive approach to the use of antibiotics in COVID-19 patients, as it quickly became apparent that COVID-19 patients do not benefit from taking antibiotics, as bacterial co-infections and secondary infections are not as common as originally thought [11,12,13]. In practice, this meant that antibiotics were not prescribed even for patients with high serum biomarkers for infection, such as C-reactive protein or procalcitonin, as the specificity of the two was not good enough to predict secondary bacterial pneumonia in COVID-19 patients [14,15,16]. Overall, this could be reflected in the lower incidence density of MDR infections against the background of the increasing MDR burden as in our case [17,18]. On the other hand, a higher incidence density of MDR burden is of concern for most MDR, but especially for Gram-negative MDR, as colonized patients pose a risk of developing MDR infection themselves and transmitting MDR to other hospitalized patients [18]. Our data on MDR burden also clearly show that active surveillance and contact precautions could be effective strategies to contain MDR in our hospital. As in the case of VRE-FA, ESBL-KP and CRAB, a temporary increase in the burden of these organisms did not lead to a sustained increase in the infection density of MDR infections.

Two meta-analyses were recently published in an attempt to determine the impact of the COVID-19 pandemic on AMR [9,10]. Langford et al. included 23 studies, most of which were from hospital settings. They found that the pandemic was not associated with a change in incidence density or proportion of MDR Gram-positive organisms. At the same time, there was a non-statistically significant increase in the subset of studies that reported the incidence density of MDR Gram-negative bacteria [9]. Abubakar et al. included 30 studies, most of which were from Italy, and found an increase in the rate of Gram-positive and Gram-negative bacteria during the pandemic, while ESBL-producing Enterobacteriaceae and CRPS decreased during that time [10]. The studies from both meta-analyses used different metrics: either the incidence density or the proportion of MDR organisms, and they were compared in a quasi-experimental design or in an interrupted time series analysis. In addition, there was a heterogeneous approach to the inclusion of bacteria from clinical or surveillance cultures, which made comparison between studies difficult. In our study, we decided to use the incidence density of clinical and combined clinical/surveillance cultures separately to better determine the effects of each factor on AMR development over time. Based on the subset of studies by Abubakar et al. [10] that used the same metrics, our clinical setting may be positioned among those with a lower AMR problem.

Compared to the published data from Slovenia, which originate from a similar study in a tertiary care hospital, the incidence density of infections with *S. aureus*, *E. faecium* and *P. aeruginosa* was increased in both clinical settings [19]. But there are still some differences. The first drastic difference was the lower absolute incidence density of MDR burden in the study, which ranged from 93.5 to 121.7 per 1000 patient days in the period before and during COVID-19, much higher than in our study [19]. This could be explained by the difference in the type of hospital (i.e., secondary vs. tertiary care) and by the fact that our hospital was not as affected by COVID-19-related hospitalizations (i.e., <10%). In general, the use of antimicrobials can be better controlled in smaller (i.e., secondary care) hospitals [20]. Nonetheless, similar trends of increase in MDR burden were observed for most MDR in both hospitals, with the exception of MRSA, which decreased in both hospitals. This similarity can be explained by the fact that both hospitals are from the same country (i.e., the same medical system) and patients are sometimes transferred between the two hospitals due to the escalation or de-escalation of treatment. The second difference between the two data sets is the direction of change in overall MDR infections, as they observed an increase from 33.2 to 54.1 per 1000 patient days, in contrast to our statistically significant decrease from 1.61 to 1.29 per 1000 patient days [19]. However, this could be due to the differences in the structure of hospitalized patients, the frequency of microbiological sampling and probably some other factors. We did not specifically calculate the incidence of MDR bloodstream infections as our numbers were too low to draw any conclusions.

There are several surveillance systems for AMR monitoring. One of the most complete and valuable in Europe is EARS-Net [21]. It reports the resistance proportions of certain organism–antibiotic combinations that are of epidemiological importance, most of which were also included in our study. EARS-Net surveillance focuses only on blood culture isolate data, so comparisons with our study must be subtle. Nevertheless, a comparison between the 2018 and 2022 EARS-Net data shows a decreasing trend for MRSA, ESBL-EC and ESBL-KP, a stable trend for CRPS and CRAB and an increasing trend for VRE-FA and CRE (i.e., carbapenem-resistant *K. pneumoniae*). This is also true for our data on MDR infections. However, we also included isolates from other infection sites. Fortunately, we have not yet seen an increase in CRE infections in our hospital, as is the case in some Slovenian hospitals, but we are currently observing an increase in CRE colonization.

Our study has several limitations. First, our data could be influenced by the sampling strategy, both for clinical and surveillance cultures. However, as far as we know, the sampling strategies for both types of culture did not change over time and were based on the presence of clinical syndrome in clinical samples and the presence of risk factors in surveillance cultures. Second, this study was performed in a low AMR clinical setting, so the impact of the pandemic could be different than in a high AMR setting. Finally, there were relatively few COVID-19-related hospitalizations in the hospital during the pandemic, so more controlled infection prevention and control measures could be implemented. Nevertheless, we were able to combine the microbiological and hospital data over a considerably long period to better understand the trends and pathways of AMR in this clinical setting.

When considering the implications, we must be cautious and not interpret the decline in MDR infections as an excuse to reduce surveillance, but rather the opposite. Due to the increasing burden of MDR, it is necessary to strengthen AMR surveillance. In addition, strict IPC measures and antimicrobial stewardship programs continue to be important components in the fight against resistant bacteria.

## 4. Materials and Methods

### 4.1. Study Design and Clinical Setting

This was a retrospective cohort observational study conducted at Izola General Hospital, a secondary care teaching hospital in the Coastal–Karst region of Slovenia, close to the border with Italy and Croatia. The hospital serves a population of 170,000 inhabitants and has 300 acute beds, with approximately 15,000 admissions per year and around 63,000 patient days.

During the COVID-19 period, the hospital was organized as a mixed-type hospital, serving both COVID-19 and non-COVID-19 patients. During 2020, 2021 and 2022, 5.2%, 9.5% and 5.1% of patient days accounted for COVID-19 patients, respectively. Based on the demand, COVID-19 units were opened in several hospital wards, including an intensive care unit. Medical teams working in the COVID-19 units were specially trained in IPC techniques and followed the hospital’s and national bodies’ evolving IPC recommendations.

In terms of general IPC capacity, the hospital is divided into five-bed and two-bed rooms, which can be converted into isolation rooms for individuals or cohorts if required. The intensive care unit has twelve beds, four of which are in single-bed rooms.

The study period was from 2017 to 2023. The pre-COVID-19 period was defined as from the start of this study until March 2020. The COVID-19 period was defined as from April 2020 to the end of the study period.

### 4.2. Data Collection

Microbiological data were collected from the laboratory information system (MBL, SRC Infonet, Naklo, Slovenia). The results of two types of bacterial cultures were extracted. Firstly, surveillance cultures were taken from patients on admission and during hospitalization, according to the hospital’s protocol based on the presence of risk factors. They selectively targeted the detection of all major MDR. Secondly, clinical cultures were taken during the diagnostic work-up of the patient, targeting clinically significant bacteria including MDR organisms. The MDR organisms included in this study were MRSA, VRE-FA, ESBL-EC, ESBL-KP, CRE, CRPS and CRAB.

*MDR burden* was defined as the isolation of a specific MDR organism in surveillance and clinical cultures per patient. MDR burden includes both colonization isolates and clinically significant isolates. Environmental culture results were excluded from this calculation. *MDR infection* was defined as the isolation of a specific MDR organism exclusively in clinical cultures per patient. Both were expressed and normalized per 1000 patient days (i.e., incidence density).

Hospital data were extracted from the hospital information system (Birpis, SRC Infonet, Slovenia). Patient days were calculated from the number of hospitalizations and length of stay for monthly intervals.

### 4.3. Microbiological Method

The bacterial isolates were identified using the MALDI Biotyper (Bruker Daltonics, Bremen, Germany). Antimicrobial susceptibility testing and resistance phenotype detection were primarily performed using the disc diffusion method according to the latest EUCAST guidelines [22]. Briefly, the MRSA phenotype was detected with a cephoxitin screening disc, the VRE phenotype was detected with a vancomycin disc after 24 h of incubation, and ESBL production was confirmed with either a combined disc method using cefotaxime, ceftriaxone and co-amoxiclav discs or with a double-disc synergy test using cefotaxime, ceftazidime and cefepime discs with and without clavulanic acid [23]. The CRE phenotype was detected by disc diffusion screening using meropenem and ertapenem discs and confirmed by antigen/molecular testing for the presence of the most common carbapenemases (i.e., KPC, VIM, IMP, NDM and OXA-48). The CRPS phenotype was defined as simultaneous resistance to carbapenems, cephalosporins and penicillin. The CRAB phenotype was defined as resistance to carbapenems.

### 4.4. Statistical Analysis

Two types of before/after analyses were performed using the statistical package JASP version 0.19.0 (University of Amsterdam, Amsterdam, The Netherlands). First, we performed the Mann–Whitney U-test to test whether there was a difference between the normalized monthly incidence densities in the two periods (pre-COVID-19 and COVID-19). Statistical significance was set at a *p*-value of 0.05. The rank biserial correlation and the corresponding 95% confidence interval expressed the effect size.

Second, we performed an interrupted time series analysis of trends on the normalized monthly MDR burden/infection incidence data using the Prophet statistical model [24]. This is a non-experimental statistical method that compares two data sets and takes into account trends, seasonality and time of the data points. The breakpoint between the two periods was set at April 2020. The growth rate (k) and offset (m) were calculated for the pre-COVID-19 period and the change in growth rate (δ) was calculated for the COVID-19 period with the corresponding 95% confidence intervals.

Finally, the MDR burden/infection was visualized for all MDR as a group and for the specific MDR in a timeline and, if applicable, a non-MDR infection was plotted as a reference point.

## 5. Conclusions

We report that in a hospital setting with a low burden of COVID-19-related hospitalizations, the COVID-19 pandemic did not worsen the incidence of MDR-related infections. On the contrary, for the majority of observed MDR and overall, infections either remained equal or significantly decreased. Nevertheless, a worrying increase in the MDR burden of most of the monitored MDR was observed, among which the most worrying is the rise of carbapenem-resistant bacteria, particularly CRE and CRPS. Finally, the two statistical methods provided comparable results for the comparison of the two time periods.

## Figures and Tables

**Table 1:** Comparison of incidence density of MDR burden and infections in the pre-COVID-19 and COVID-19 periods using the Mann–Whitney U-test and interrupted time series analysis.

	Mann–Whitney U-Test					Interrupted Time Series Analysis		
	Pre-COVID Incidence Density Mean	COVID Incidence Density Mean	*p*-Value	Rank Biserial Correlation (RBC) ^1^	95% Confidence Interval of RBC	Pre-COVID Incidence Density Offset (m)	Pre-COVID Incidence Density Growth Rate (k)	COVID Incidence Density Growth Rate Change (δ)
**MDR Burden**								
**ALL MDR**	**4.93**	**5.81**	**0.007**	**−0.34**	**(−0.54**, **−0.11)**	**0.466**	**0.151**	**−0.062**
MRSA	1.45	1.21	0.067	0.23	(−0.01, 0.45)	0.485	−0.124	−0.005
VRE-FA	0.23	0.59	**0.001**	−0.38	(−0.57, −0.15)	0.048	0.130	−0.128
ESBL-EC	2.26	2.65	**0.022**	−0.29	(−0.50, −0.05)	0.518	0.185	−0.024
ESBL-KP	0.56	0.81	**0.015**	−0.31	(−0.52, −0.07)	0.192	0.083	0.026
CRE	0.11	0.19	0.055	−0.22	(−0.44, 0.02)	0.043	0.278	0.027
CRPS	0.11	0.20	**0.023**	−0.27	(−0.48, −0.02)	0.083	0.307	−0.006
CRAB	0.10	0.02	0.062	0.16	(−0.09, 0.39)	0.144	−0.139	0.013
**MDR Infections**								
**ALL MDR**	**1.61**	**1.29**	**0.019**	**0.30**	**(0.06**, **0.51)**	**0.548**	**−0.183**	**0.027**
MRSA	0.32	0.30	0.384	0.11	(−0.14, 0.35)	0.262	−0.011	−0.014
VRE-FA	0.02	0.03	0.258	−0.08	(−0.32, 0.17)	0.050	0.117	−0.024
ESBL-EC	0.79	0.51	**<0.001**	0.43	(0.21, 0.61)	0.531	−0.281	0.038
ESBL-KP	0.27	0.28	0.462	−0.09	(−0.33, 0.16)	0.217	−0.043	0.036
CRE	0.06	0.02	**0.039**	0.16	(−0.09, 0.39)	0.088	−0.056	0.010
CRPS	0.06	0.11	0.085	−0.18	(−0.41, 0.07)	0.066	0.197	−0.003
CRAB	0.05	0.01	**0.008**	0.18	(−0.07, 0.41)	0.120	−0.121	−0.009

^1^ Rank biserial correlation (RBC) is an effect size for the Mann–Whitney U-test. The values ±0.1, ±0.3, and ±0.5 and above correspond to small, medium and large differences between the two groups in both directions.

## Data Availability

Data are contained within the article.
